# The relationship between amino acids and gastroesophageal reflux disease: evidence from a mendelian randomization analysis combined with a meta-analysis

**DOI:** 10.3389/fimmu.2025.1420132

**Published:** 2025-03-04

**Authors:** Jianjun Shen, Yongqing Guo, Rui Cao

**Affiliations:** ^1^ Jiamusi College, Heilongjiang University of Chinese Medicine, Jiamusi, China; ^2^ Capital University of Physical Education and Sports, Beijing, China

**Keywords:** amino acids, gastroesophageal reflux disease, the omics mendelian randomization, meta analysis, authenticate reverse, multiple corrections

## Abstract

**Background:**

Gastroesophageal Reflux Disease (GERD), a prevalent gastrointestinal disorder globally, exhibits variable prevalence across regions, with higher frequencies observed in Western nations and lower in Asian countries. Key contributing factors encompass unhealthy eating patterns, tobacco use, consumption of alcohol, excess weight, and obesity, along with health conditions such as gestation and diabetes. Common manifestations include heartburn and a burning discomfort behind the breastbone, which, without appropriate management, can progress to more severe issues like esophagitis and Barrett’s esophagus. Approaches to management and prevention primarily involve modifications in lifestyle, pharmacotherapy, and surgical interventions when deemed necessary. Utilizing Omics Mendelian Randomization (OMR) to investigate the causative links between genetic variants and diseases provides insights into the biological underpinnings of gastroesophageal reflux diseasec. It aids in pinpointing novel targets for therapy. The influence of amino acids in gastroesophageal reflux disease demonstrates the complexity, having the potential to both mitigate and intensify symptoms, underscoring the significance of personalized nutrition and therapeutic strategies.

**Methods:**

This study is based on the omics mendelian randomization method, coupled with meta-analysis techniques, to enhance the precision of the research findings. Furthermore, a reverse validation procedure was implemented to validate the association between the positive findings and disease outcomes further. Throughout the study, multiple correction measures were employed to ensure the accuracy and reliability of the results.

**Results:**

Based on our research methodology, we have ultimately discovered that glutamate exacerbates gastroesophageal reflux disease, increasing its risk. The data supporting this includes analysis of 20 amino acids and outcomes from the Finnish database, which showed that glutamate had an odds ratio (OR) for gastroesophageal reflux disease risk of 1.175(95% confidence interval (CI): 1.000 ~ 1.380, P = 0.05), and a beta value of 0.161. Analysis with outcomes from the UK database indicated that glutamate had an OR for gastroesophageal reflux disease risk of 1.399(95% CI: 1.060 ~ 1.847, P = 0.018) and a beta value of 0.336. After conducting a meta-analysis of the MR results and applying multiple corrections, the combined OR of glutamate for gastroesophageal reflux disease risk was 1.227 (95% CI: 1.068 ~ 1.411 P = 0.043); the beta values of the three primary MR outcomes were consistent in direction. Building on the positive results, reverse validation with outcome data from two different database sources for glutamate showed: in the Finngen database, with gastroesophageal reflux disease as the exposure, the Inverse Variance Weighted (IVW) method resulted in a P-value of 0.059; in the IEU database under the same condition, the IVW P-value was 1.433.

**Conclusions:**

Glutamate may increase the risk and exacerbate the progression of gastroesophageal reflux disease through mechanisms such as impacting the nervous system and promoting inflammatory responses. Delving into the role of glutamate in gastroesophageal reflux disease enriches our understanding of the disease’s biological mechanisms and may offer new strategies for clinical treatment and nutritional management. This insight can aid in developing healthier dietary plans, thereby benefiting patients.

## 1 Introduction

Gastroesophageal reflux disease (GERD) is a globally prevalent gastrointestinal disorder, with an incidence rate of approximately 10% to 20% among adults in Western countries, while being relatively lower in Asia, at around 5%. Major risk factors for gastroesophageal reflux disease include unhealthy dietary habits (e.g., high-fat and high-calorie food intake), smoking, alcohol consumption, overweight and obesity, as well as metabolic conditions such as diabetes. The hallmark symptoms of gastroesophageal reflux disease are acid regurgitation and retrosternal burning sensation. Some patients may also present with atypical symptoms, such as dysphagia, chronic cough, and hoarseness. If left untreated, gastroesophageal reflux disease can lead to serious complications, including esophagitis, esophageal strictures, Barrett’s esophagus, and even esophageal adenocarcinoma. Notably, Barrett’s esophagus, a precancerous condition, is closely associated with chronic irritation and inflammation caused by gastroesophageal reflux disease, warranting significant clinical attention (1, 2).

In terms of prevention and management, gastroesophageal reflux disease treatment typically begins with lifestyle modifications, such as avoiding high-fat and highly acidic foods, reducing smoking and alcohol consumption, managing body weight, and refraining from lying down immediately after meals. Pharmacological therapy remains the primary intervention, with proton pump inhibitors (PPIs) and H2 receptor antagonists effectively alleviating symptoms and promoting esophageal mucosal healing by reducing gastric acid secretion. For severe cases or those unresponsive to medication, surgical interventions such as fundoplication may be required. Understanding the epidemiological characteristics and diverse risk factors of gastroesophageal reflux disease is crucial for developing precise prevention and treatment strategies. Furthermore, continued research into its etiology and pathogenesis will provide a scientific foundation for improving disease management and mitigating associated complications (3–5).

The role of amino acids in the management of gastroesophageal reflux disease is dual in nature. On one hand, certain amino acids, such as glutamine, may positively contribute to symptom relief by supporting gastric mucosal repair and protection. On the other hand, some amino acids, such as histidine, may exacerbate gastroesophageal reflux disease symptoms by stimulating gastric acid secretion. Additionally, high-protein diets can prolong gastric emptying time, increasing the likelihood of reflux and potentially aggravating symptoms in some cases. Individual responses to amino acids and proteins are highly personalized, as specific foods or nutrients may trigger symptoms in some individuals but have no apparent impact in others. Therefore, it is crucial for patients with gastroesophageal reflux disease to adopt personalized dietary and treatment plans under the guidance of healthcare professionals to effectively manage their symptoms (6, 7).

Current research on the relationship between amino acids and gastroesophageal reflux disease remains in the exploratory phase, primarily focusing on metabolomics and observational studies. Evidence suggests that amino acids may influence the pathogenesis of gastroesophageal reflux disease through mechanisms such as regulating gastrointestinal acid-base balance, enhancing gastric mucosal barriers, and modulating inflammation and oxidative stress. For instance, glutamine has been found to aid in the repair of gastroesophageal mucosa, while abnormalities in branched-chain amino acids and specific amino acid metabolites may be associated with gastroesophageal reflux disease-related inflammatory responses. However, most existing studies are limited by small sample sizes, predominantly conducted in Western countries, and focus mainly on correlation rather than causation. Animal experiments have indicated potential protective effects of amino acids, but large-scale clinical trials to substantiate these findings are lacking. Furthermore, current research pays little attention to racial and genetic diversity, and mechanistic studies remain limited (8, 9).

Mendelian randomization studies have emerged as a valuable tool for uncovering causal relationships in gastroesophageal reflux disease. Existing Mendelian randomization research, leveraging genetic instrumental variable analysis, has confirmed that high body mass index and smoking are significant risk factors for gastroesophageal reflux disease. Additionally, Mendelian randomization studies have explored the causal links between gastroesophageal reflux disease and metabolic disorders such as diabetes and hypertension, as well as related conditions like Barrett’s esophagus and esophageal adenocarcinoma. Mendelian randomization studies have also highlighted the potential roles of inflammatory factors and genetic variations in gastroesophageal reflux disease pathogenesis, offering new biological insights into disease mechanisms. With the accumulation of large-scale genome-wide association study data, Mendelian randomization methods show great potential for elucidating the causes of gastroesophageal reflux disease and guiding precise prevention and intervention strategies. However, no Mendelian randomization studies to date have specifically investigated the causal relationship between amino acids and gastroesophageal reflux disease, nor have meta-analyses been conducted to validate these associations (10–12).

Omics Mendelian Randomization (OMR) employs a strategy grounded in Mendelian randomization principles, utilizing genetic variants as instrumental variables to delineate causal connections between omics data (like proteins, metabolites, and microbiota) and disease outcomes. This approach substantially mitigates the issues of confounders common in observational studies, determines the causal linkage between exposure and disease, and aids in the identification and corroboration of novel biomarkers related to disease. The deployment of this methodology, particularly in complex disease research, not only enriches our comprehension of diseases’ biological underpinnings but also unveils novel targets for intervention (13–15).

Their biphasic nature characterizes the significance of amino acids in gastroesophageal reflux disease management. For instance, amino acids such as glutamine may relieve gastroesophageal reflux disease symptoms by aiding the gastric mucosa’s healing and protective mechanisms (16, 17). Conversely, amino acids like histidine might intensify GERD symptoms by enhancing gastric acid secretion. Moreover, diets high in protein could delay gastric emptying, heightening reflux chances and potentially worsening symptoms in specific scenarios. The response to amino acids and proteins varies significantly among individuals, with specific diets or nutrients triggering symptoms in some while being innocuous in others (18, 19). Consequently, individuals with GERD must pursue personalized dietary and therapeutic approaches under professional healthcare guidance to manage symptoms effectively.

Based on current observational studies and traditional two-sample Mendelian randomization studies, this research conducted omics Mendelian randomization analyses to investigate the relationships between 20 amino acids and gastroesophageal reflux disease using data from both the Finngen database and the UK Biobank database. Subsequently, meta-analysis was performed on the inverse-variance weighted (IVW) results from the two analyses, combining the findings from the two databases to achieve more precise results. This approach allows for a deeper understanding of how specific amino acids influence the development and progression of gastroesophageal reflux disease through particular biological pathways, thereby uncovering potential therapeutic targets and prevention strategies.

## 2 Materials and methods

### 2.1 Study design

**Figure 1:**
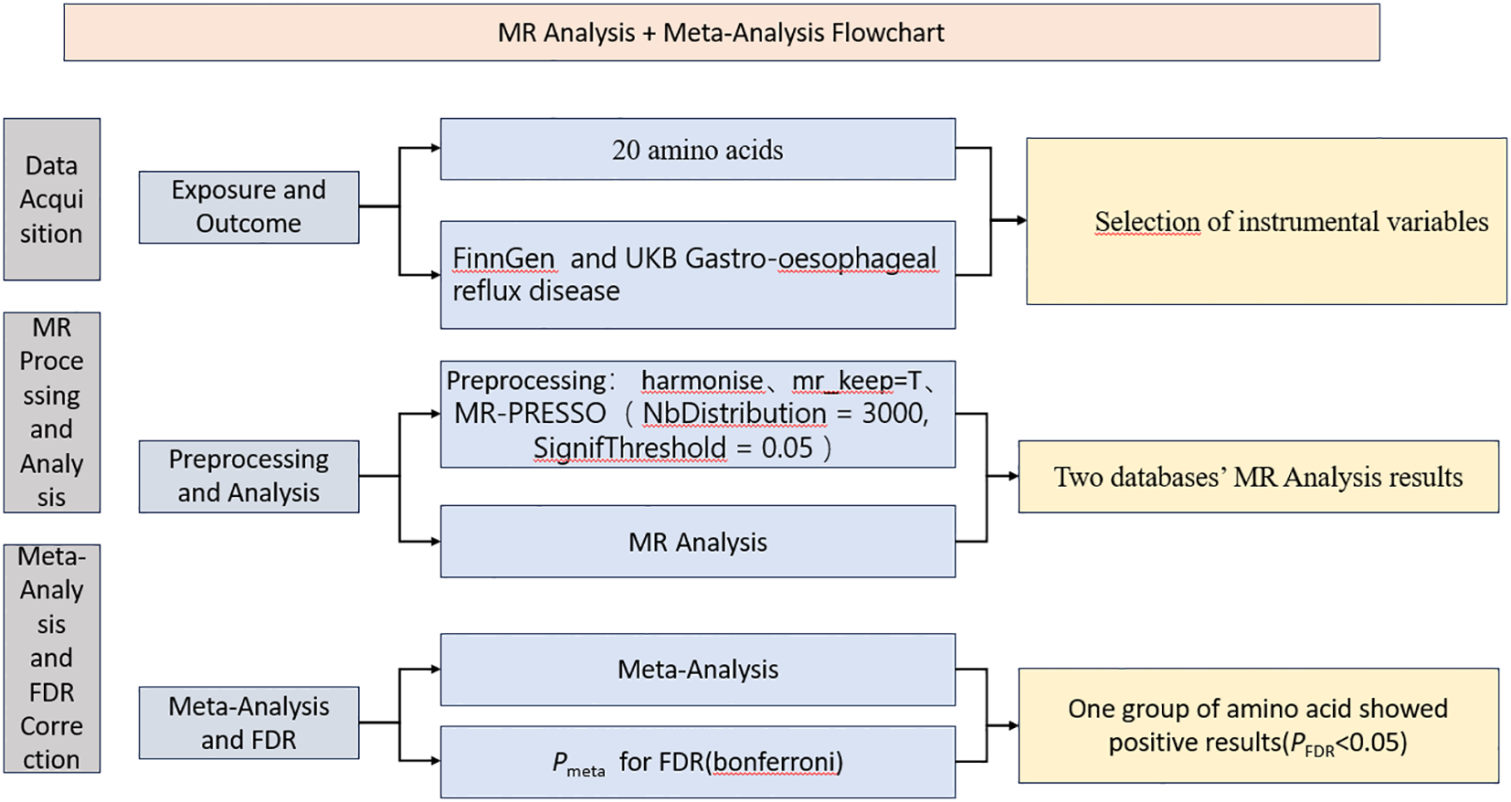
The process flowchart of the research methodology.

This study comprehensively evaluates the associations between data through three detailed phases, initially gathering critical exposure data and two independent outcome datasets and screening influential instrumental variables to lay the foundation for estimating causal relationships between variables. Subsequently, it delves into sensitivity analysis and employs the two-sample Mendelian Randomization approach to compare exposure data with the two outcome datasets, revealing potential causal links. Ultimately, synthesizing different study results through a meta-analysis of the inverse variance weighted outcomes enhances statistical power and conclusion reliability and employs multiple corrections to reduce biases and Type I errors, ensuring the rigor of the conclusions. These meticulous steps bolster the credibility of the study’s results and provide methodological references for future research. The data obtained in this study all come from public databases, and the participation behind the data has received approval from relevant institutions and committees. A flowchart was also created for this study (**Figure 1**).

This study, building on omics Mendelian randomization, performed Mendelian randomization analyses to explore the relationships between 20 amino acids and gastroesophageal reflux disease using data from the Finngen database and the UK Biobank database. Subsequently, meta-analysis was conducted on the inverse-variance weighted (IVW) results from these analyses, combining the findings from the two databases to achieve more precise results. The meta-analyzed results were further subjected to multiple corrections to reduce the risk of type I errors. For example, a recent Mendelian randomization study investigating the relationship between circulating antioxidants and the risk of coronary heart disease detailed how Mendelian randomization analyses were conducted separately for circulating antioxidants and coronary heart disease outcome data from three different databases. The IVW results from the three analyses were then meta-analyzed, and the final results did not support circulating antioxidants as protective factors for coronary heart disease (20).

### 2.2 Genome-wide association study data sources for amino acid

We analyzed two publicly available genome-wide association study (GWAS) datasets on circulating amino acid levels in European populations. The study from 2016 utilized MR metabolomics methods to analyze 123 circulating metabolic traits across 14 cohorts comprising 24,925 participants, focusing on the summary and statistical data for tyrosine, valine, alanine, leucine, isoleucine, phenylalanine, glutamine, and histidine (21). In contrast, the 2014 study screened approximately 2.1 million single nucleotide polymorphisms (SNPs) from 7,824 participants across two European cohorts for genetic analysis of 486 chemically identified metabolites (22). These metabolites were classified into 8 major metabolite groups in the KEGG database, with an additional 196 metabolites categorized as unknown (23). Specifically, our attention was on the amino acid GWAS data, including aspartate, glutamate, glycine, arginine, threonine, cysteine, proline, serine, guanidinoacetate, tryptophan, and methionine.

### 2.3 Sources of GWAS data on gastroesophageal reflux disease

Our study utilized outcome data on gastroesophageal reflux disease from two independent databases, ensuring that the outcome data were sourced differently from the exposure data. Specifically, the first data set was derived from the Finngen R10 database, which included 28,859 cases and 350,064 controls (24). The second data set originated from a UK database, encompassing 29,975 cases and 390,556 controls (Pan-UKB team. https://pan.ukbb.broadinstitute.org. 2020.). In each database, we selected the dataset with the largest sample size for this condition to ensure the accuracy and reliability of our study.

The rationale for data selection is as follows:

1. Data quality and sample size: Both the Finngen and UK Biobank databases are publicly accessible and of high quality, encompassing large-scale case-control datasets. These databases provide robust statistical power and a solid foundation for Mendelian randomization analyses.
2. High prevalence of gastroesophageal reflux disease: Finland and the United Kingdom are regions with a high prevalence of gastroesophageal reflux disease, which is closely associated with the potential effects of amino acid metabolism examined in this study. Utilizing these databases increases the likelihood of identifying causal relationships.
3. Data consistency: Finngen and UK Biobank adhere to rigorous data collection and quality control protocols, ensuring high consistency and comparability of data, which strengthens the reliability of the meta-analysis results.

We acknowledge that this study did not include data from Asian countries, where the prevalence of gastroesophageal reflux disease is lower. This limitation may restrict the generalizability of the conclusions. The exclusion is primarily due to the limited availability of gastroesophageal reflux disease-related sample data from Asian populations in current public databases, with case numbers significantly smaller than those in Finngen and UK Biobank, making it difficult to achieve the statistical power required for Mendelian randomization analysis. Future studies could validate the findings of this research by collecting high-quality data from Asian populations and exploring the impact of ethnic and geographic differences.

### 2.4 Criteria for selection of instrumental variables

All our analyses were conducted under R version 4.2.1 (https://www.r-project.org/). In our investigation, we identified independent SNPs significantly correlated with levels of circulating amino acids at a genome-wide significance threshold (P<5e-8). However, for certain amino acids, it became evident that the number of SNPs at this threshold was markedly insufficient. To tackle this issue, we modified the p-value thresholds as necessary to guarantee an adequate pool of SNPs for the analysis of each specific amino acid: the p-value threshold was adjusted to 5e-7 for asparagine, leucine, proline, and serine, set to 5e-6 for arginine, cysteine, glycine, isoleucine, methionine, and threonine; and further altered to 5e-5 for glutamate and aspartate. These modifications ensured the study encompassed all selected amino acids, with enough SNPs for a thorough analysis (25, 26). We computed the F-statistics and retained the data with F greater than 10 (**Supplementary Table 1**).

## 3 Statistical analysis

### 3.1 The causal relationship between the 20 amino acids and gastroesophageal reflux disease

To enhance the efficiency and accuracy of SNP data processing for gastroesophageal reflux disease, we first selected SNP data with matching rsids from the outcome and exposure data across two distinct databases, retaining these matches for further analysis. Subsequently, we prepared the retained data for Mendelian randomization studies to ensure suitability for subsequent analysis. Further exposure and filtered outcome data were cleaned, including eliminating palindromic SNPs using the parameter action=2. Ultimately, we removed data flagged as mr_keep=false to ensure that only data contributing positively to the research were included in the final analysis set (27, 28). Through these optimization steps, we enhanced data processing efficiency and ensured the accuracy and reliability of our analysis results, providing solid data support for in-depth research on gastroesophageal reflux disease.

Building on the Mendelian randomization analysis of gastroesophageal reflux disease SNP data, we further employed MR-PRESSO to improve the analysis’s accuracy and reliability. Initially, through horizontal pleiotropy tests (**Supplementary Table 2**), we assessed the potential impacts of SNPs on multiple phenotypes to identify pleiotropic SNPs that might affect analysis accuracy. For data identified with significant pleiotropy (p-value < 0.05), we conducted in-depth processing using MR-PRESSO, setting NbDistribution to 3000 and SignifThreshold to 0.05 to precisely identify and eliminate outliers (29). These optimization measures effectively cleaned the data and ensured our analysis results were more reliable, providing a solid data foundation for revealing the complex relationship between genetic mechanisms and environmental exposure factors in gastroesophageal reflux disease.

**Figure 2:**
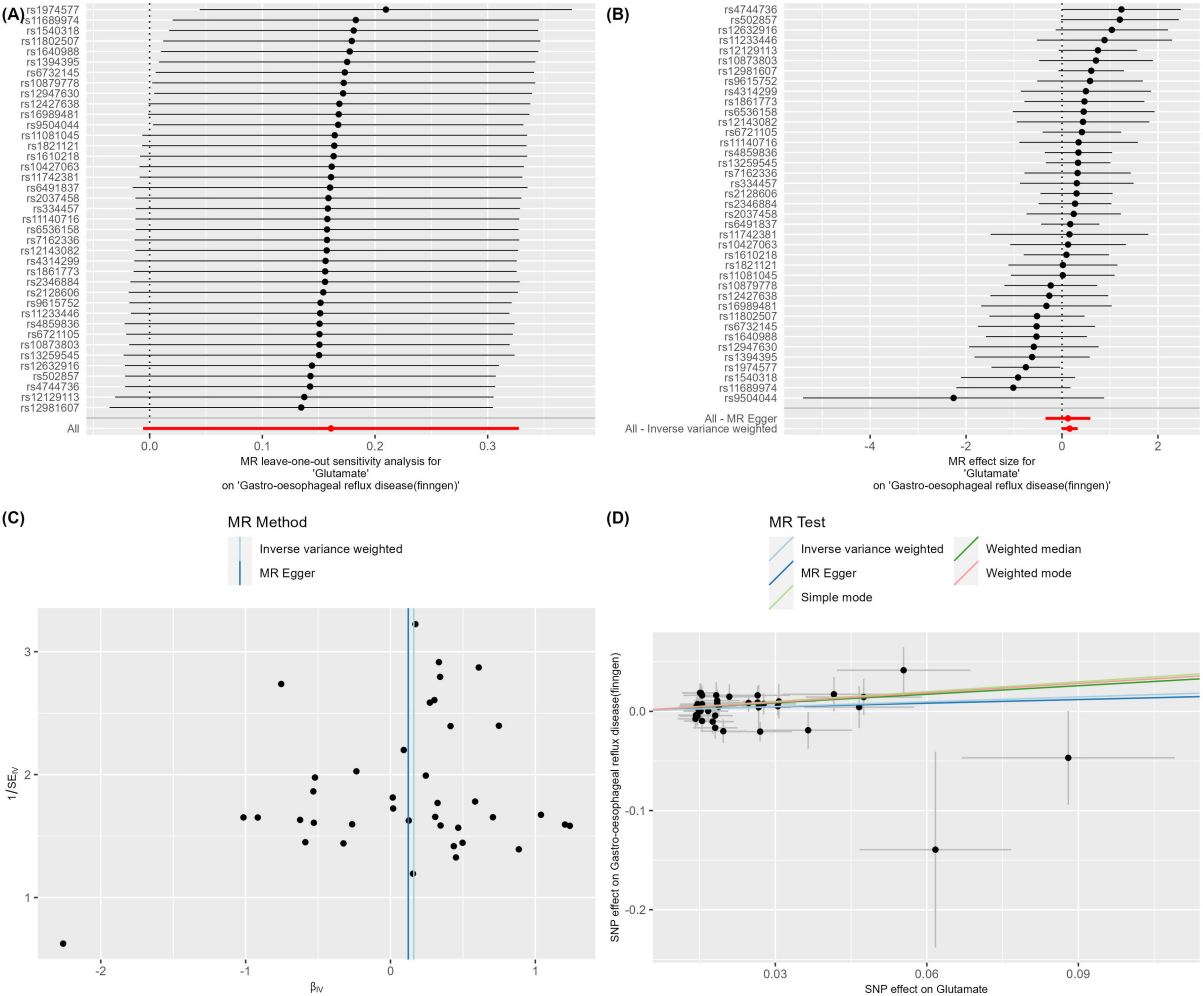
**(A–D)** Glutamate on Gastro-oesophageal reflux disease (finngen).

**Figure 3:**
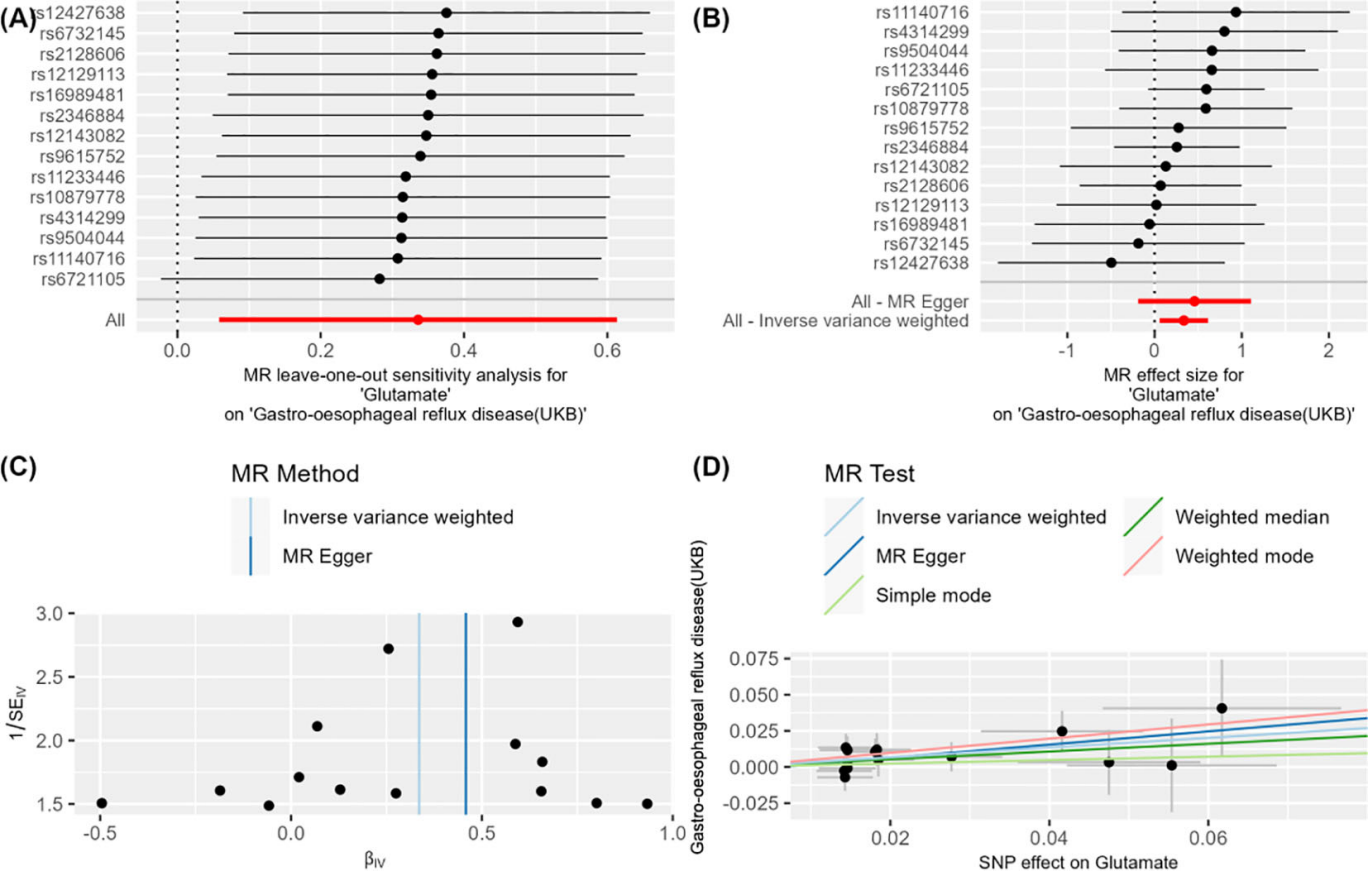
**(A–D)** Glutamate on Gastro-oesophageal reflux disease (UKB).

After completing the preprocessing and MR-PRESSO outlier removal for gastroesophageal reflux disease SNP data, we entered the final phase of MR analysis to explore the impact of genetic variants on disease risk. Initially, heterogeneity tests ((**Supplementary Table 3**)) assessed the consistency among selected SNPs, providing a basis for selecting subsequent analysis models. When significant heterogeneity was detected (Q_pval < 0.05), we used the IVW random-effect model, suitable for assuming a consistent effect of all SNPs on disease risk; in the absence of significant heterogeneity, we employed the IVW fixed-effect model, allowing for differences in SNP effect sizes (30, 31). We focused on the results of IVW, MR-Egger, and weighted median methods to comprehensively assess the relationship between genetic variations and gastroesophageal reflux disease risk. We calculated the corresponding OR to quantify the impact strength of genetic variations on disease risk. This optimized process not only improved the accuracy and reliability of MR analysis but also provided a solid scientific basis for revealing the role of genetic factors in the pathogenesis of gastroesophageal reflux disease (**Supplementary Table 4**). In addition, we also created a composite figure of the MR results (**Figures 2**, **3**).

**Figure 4:**
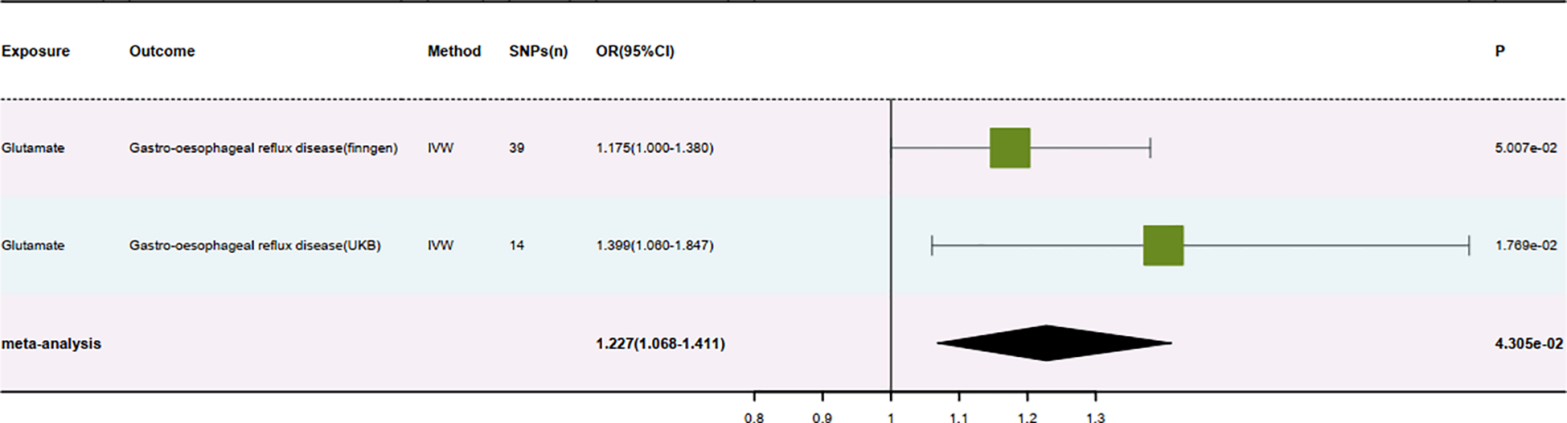
Result forest map.

We conducted a meta-analysis of IVW results from the two previous MR analyses to verify the robustness of the results through the synthesis of different outcome data (**Supplementary Table 5**). To minimize the false-positive rate, we implemented multiple correction measures on the meta-analysis results, ensuring high precision of the outcomes. We also created a forest plot of the final positive results following the meta-analysis (**Figure 4**).

### 3.2 The causal association between gastroesophageal reflux disease and positive amino acids

We conducted a positive analysis by identifying the amino acids as outcome data while considering the original outcome data—gastroesophageal reflux disease—as the exposure factor. We also set a P-value threshold of 1E-5 to ensure the strictness of the screening criteria. Through this method, we aimed to explore whether gastroesophageal reflux disease could lead to changes in amino acid levels. However, the analysis did not find a significant reverse causal relationship (31, 32). Our understanding of the relationship between gastroesophageal reflux disease and amino acid levels was deepened using a reverse validation perspective. Although no direct reverse causal link was discovered, this process enhanced our recognition of the disease’s complexity and provided new perspectives for future research directions (**Supplementary Table 6**).

## 4 Results

### 4.1 The causal relationship between the 20 amino acids and gastroesophageal reflux disease

Our study explored the potential causal relationship between specific amino acids and gastroesophageal reflux disease (GERD) through a series of Mendelian randomization analyses combined with meta-analysis and multiple corrections. The results indicated a significant causal link only between glutamate and gastroesophageal reflux disease (**Supplementary Table 7**). Specifically, analysis from the Finnish database showed that the OR for glutamate concerning the risk of gastroesophageal reflux disease was 1.175 (95% CI: 1.000-1.380, P = 0.05) and a beta value of 0.161. Analysis from the UK database showed an OR for glutamate concerning gastroesophageal reflux disease of 1.399 (95% CI: 1.060-1.847, P = 0.018) and a beta value of 0.336. After meta-analysis and FDR correction, the pooled OR for glutamate about gastroesophageal reflux disease was 1.227, with a 95% CI: 1.068-1.411, P = 0.043). These results consistently indicate a positive association between increased levels of glutamate and a higher risk of gastroesophageal reflux disease, with the direction of the beta values under all three methods in the MR analysis from both databases consistently showing an exacerbation of gastroesophageal reflux disease and an increased disease risk.

This research, through precise statistical methods and stringent correction measures, enhanced the reliability and interpretability of the results. The causal relationship between glutamate and gastroesophageal reflux disease reveals new biological mechanisms, potentially offering new targets for future therapeutic strategies. Moreover, this finding also emphasizes the importance of considering specific metabolite levels in disease risk assessment and management. Future studies could further explore the role of glutamate in the pathogenesis of gastroesophageal reflux disease and how regulating glutamate levels may prevent or treat gastroesophageal reflux disease.

### 4.2 The causal association between gastroesophageal reflux disease and positive amino acids

In addressing gastroesophageal reflux disease, we utilized glutamate as the exposure factor in our approach and conducted a two-sample MR analysis. Furthermore, we applied multiple corrections to ensure the accuracy of our findings. The analysis indicated that in the Finngen database, with gastroesophageal reflux disease as the exposure factor, the P-value from the IVW method was 0.059. In contrast, in the UKB database, under the same condition, the IVW P-value was 1.433.

Based on the current analysis, these results suggest no reverse causal relationship between glutamate and gastroesophageal reflux disease. In other words, we did not find sufficient evidence to support that gastroesophageal reflux disease could influence the metabolism and function of amino acids. This discovery helps us deepen our understanding of the relationship between gastroesophageal reflux disease and glutamate, providing crucial information for future research directions and treatment strategies.

## 5 Discussion

Gastroesophageal Reflux Disease (GERD), a prevalent digestive system disorder, is principally characterized by the backflow of stomach acids or gastric contents into the esophagus, causing symptoms and complications. Significant symptoms of gastroesophageal reflux disease include heartburn, discomfort in the throat, chest pain, and others. Persistent reflux may lead to complications such as inflammation of the esophagus, narrowing of the esophagus, and Barrett’s esophagus. Glutamate, a critical neurotransmitter involved in many physiological functions, including the modulation of gastrointestinal functions, has gained attention in studying gastroesophageal reflux disease, especially its role within the gastrointestinal neural system, which could significantly influence the disease’s pathogenesis and potential treatment options (33, 34).

Glutamate enhances gastrointestinal motility by activating receptors within the gastrointestinal neural system, notably NMDA and non-NMDA receptors. This increased motility may result in temporary relaxation of the lower esophageal sphincter (LES), a normal physiological response that allows the passage of food from the esophagus to the stomach (35, 36). However, in individuals with gastroesophageal reflux disease, such relaxation of the LES might occur excessively or inappropriately, leading to the backflow of gastric contents into the esophagus. Furthermore, glutamate may impact the gastric emptying rate; improper acceleration of gastric emptying could heighten the pressure on the LES, further facilitating reflux.

Research into the physiological role of dietary L-glutamate (Glu) during the gastric phase delves into how glutamate, the only amino acid typically ingested in its free form, significantly boosts gastric secretion and motility, particularly with diets rich in proteins and amino acids. This action is closely linked to the stimulation of vagal afferent fibers, driven by a nitric oxide-led paracrine cascade and serotonin (5-HT) involvement, affecting 5-HT3 receptors, highlighting its importance in regulating gastric digestion (37–39). The role of enteroendocrine G and D cells in assimilating amino acid-induced signals further underscores glutamate’s crucial role in maintaining digestive health.

Glutamate plays a multifaceted role in promoting inflammatory responses, directly stimulating immune cells, and enhancing the transmission of inflammatory signals through neuro-immune interactions. Within the esophageal mucosa, for instance, glutamate can directly activate macrophages and lymphocytes, producing pro-inflammatory cytokines and chemokines. This attracts more immune cells, exacerbating the local inflammatory response and potentially causing further tissue damage. Moreover, glutamate’s role in neuro-immune communications suggests that it can activate distant immune cells via neural pathways, extending the inflammatory response systemically beyond the initial site. Understanding these mechanisms provides crucial insights into potential therapeutic interventions, such as targeting glutamate receptors on immune cells or modulating neuro-immune pathways, offering promising avenues for treating inflammatory conditions more effectively (40, 41).

Investigations into immune responses in neurological disorders associated with anti-glutamic acid decarboxylase antibodies concentrated on both inflammatory and anti- Investigations into brain disorders related to antibodies against glutamic acid decarboxylase unveil a nuanced disruption in the equilibrium between immune activation and suppression. These inquiries reveal a significant diminution in the populations of immune regulatory cells, which is crucial for sustaining immune tolerance and averting autoimmune phenomena. Such a decline indicates a compromised capacity to regulate immune activation, potentially leading to unbridled inflammatory conditions. Simultaneously, an escalation in intermediate monocytes, known for their pro-inflammatory roles, suggests an intensified state of inflammation. This skewed balance could amplify the neurological manifestations associated with these disorders, creating an unchecked, inflammation-driven environment (41–43). Strategies to rectify this imbalance through bolstering regulatory mechanisms or curbing inflammatory processes could emerge as viable therapeutic approaches to reestablish immune harmony in these diseases.

The integrity of the esophageal mucosal barrier plays a vital role in safeguarding the esophagus against erosion by stomach acid and enzymes. Glutamate may influence the integrity of this barrier by regulating the proteins that facilitate cellular connections in the submucosa, such as tight junctions and adherens junctions. When these cellular connections are compromised, the barrier function of the esophageal mucosa diminishes, allowing easier penetration by stomach acid and enzymes, leading to inflammation and damage. Moreover, glutamate may also affect the survival and regenerative capabilities of mucosal cells, further impacting the barrier function of the esophageal mucosa (44, 45).

The role of glutamate in pain perception primarily manifests through its excitatory actions within the central and peripheral nervous systems. In gastroesophageal reflux disease, damage to the esophageal mucosa caused by acid reflux may activate pain receptors in the esophagus, and glutamate could exacerbate pain perception by enhancing the signaling of these receptors. This mechanism not only involves direct effects on the nerves in the esophagus but may also include regulating pain processing pathways in the central nervous system, such as increasing the transmission and processing of pain signals in the spinal cord and brain (46).

Although glutamate’s primary function is as a neurotransmitter, it may also indirectly affect gastric acid secretion through neuro-endocrine pathways. Here, neurons activated by glutamate can influence the release of hormones like gastrin that regulate gastric acid secretion. Gastrin, an essential hormone for gastric acid secretion regulation, stimulates acid production by acting on parietal cells in the stomach wall. Consequently, glutamate might indirectly affect gastric acid production by modulating the release of gastrin, thereby influencing the progression and symptoms of gastroesophageal reflux disease (47).

Examining the pivotal function of esophageal afferent nerves in gastroesophageal reflux disease thoroughly investigated their importance in developing gastroesophageal reflux disease symptoms. This study highlighted the essential role sensory nerves within the esophagus play, particularly their influence on pain perception in individuals afflicted with gastroesophageal reflux disease. The investigation pointed out the critical nature of acid-sensitive receptor presence on these nerves for understanding the neurophysiological foundations of heartburn. The research found that differences in the expression and positioning of submucosal afferent nerves among various gastroesophageal reflux disease phenotypes might elucidate how patients with comparable levels of reflux experience symptoms. Additionally, the study explored how both central and peripheral sensitization pathways could magnify or diminish the conveyance of incoming signals by adjusting the intensity of activation of sensory nerves in the esophagus. Such a mechanism might account for the variation in pain perception observed in gastroesophageal reflux disease sufferers (48, 49). This analysis provides a fresh viewpoint on deciphering the intricate mechanisms behind pain perception in the esophagus, focusing on the neurophysiological aspects of gastroesophageal reflux disease symptoms.

Based on numerous observational studies, this research validates the causal relationship between 20 amino acids and gastroesophageal reflux disease at the genetic level, achieving a fully randomized controlled trial at the genetic level. This approach mitigates confounding factors inherent in observational studies, providing a more precise understanding of the relationship between the two. The combination of Mendelian randomization analysis and meta-analysis enhances the reliability and credibility of the findings compared to single analyses. However, the study has certain limitations. Due to restrictions in data sources, the sample population in the research is predominantly of European ancestry, which may limit the generalizability of the results to global populations. Future studies should expand to include other ethnicities and regions to further validate and extend these findings. Despite these limitations, this study provides a scientific basis for further research on the role of glutamate in gastroesophageal reflux disease risk and its clinical applications. Regulating glutamate levels scientifically could significantly improve public health and reduce the incidence of gastroesophageal reflux disease.

## 6 Conclusions

Glutamate plays a complex and significant role in the pathogenesis of gastroesophageal reflux disease. Studies have indicated that glutamate may facilitate the development of gastroesophageal reflux disease by activating specific neural pathways and exacerbating inflammatory responses, thereby elevating the risk of the disease and propelling its progression. This discovery has deepened our understanding of the biological foundations of gastroesophageal reflux disease and highlighted the potential importance of glutamate in disease management. Investigating the role of glutamate in gastroesophageal reflux disease allows researchers to identify new therapeutic targets and provides a scientific basis for developing targeted treatment approaches. Moreover, this research holds significant implications for the field of nutrition. Understanding how glutamate affects gastroesophageal reflux disease could enable nutritionists to offer patients more appropriate dietary guidance to minimize the adverse effects of glutamate, reduce the risk of gastroesophageal reflux disease, or alleviate existing symptoms.

## Data Availability

The datasets presented in this study can be found in online repositories. The names of the repository/repositories and accession number(s) can be found in the article/**Supplementary Material**.

## Supplementary material

Supplementary Table 1 Calculate the F-statistic for Each SNP.

Supplementary Table 2 Results of the pleiotropy test.

Supplementary Table 3 Results of the heterogeneity test.

Supplementary Table 4 Results of MR analysis.

Supplementary Table 5 Results of the Meta-Analysis.

Supplementary Table 6 Results of Reverse Validation of Positive Indicators.

Supplementary Table 7 Results after multiple corrections.
